# Proactive and integrated primary care for frail older people: design and methodological challenges of the Utrecht primary care PROactive frailty intervention trial (U-PROFIT)

**DOI:** 10.1186/1471-2318-12-16

**Published:** 2012-04-25

**Authors:** Nienke Bleijenberg, Irene Drubbel, Valerie H ten Dam, Mattijs E Numans, Marieke J Schuurmans, Niek J de Wit

**Affiliations:** 1 Julius Center for Health Sciences and Primary Care, University Medical Center Utrecht, Universiteitsweg 100, Utrecht CG 3584, The Netherlands; 2 Department of Rehabilitation, Nursing Science and Sports Medicine, University Medical Center Utrecht, Utrecht, The Netherlands

**Keywords:** Frailty, Older people, Proactive and integrated care, General practice, Primary care

## Abstract

**Background:**

Currently, primary care for frail older people is reactive, time consuming and does not meet patients' needs. A transition is needed towards proactive and integrated care, so that daily functioning and a good quality of life can be preserved. To work towards these goals, two interventions were developed to enhance the care of frail older patients in general practice: a screening and monitoring intervention using routine healthcare data (U-PRIM) and a nurse-led multidisciplinary intervention program (U-CARE). The U-PROFIT trial was designed to evaluate the effectiveness of these interventions. The aim of this paper is to describe the U-PROFIT trial design and to discuss methodological issues and challenges.

**Methods/Design:**

The effectiveness of U-PRIM and U-CARE is being tested in a three-armed, cluster randomized trial in 58 general practices in the Netherlands, with approximately 5000 elderly individuals expected to participate. The primary outcome is the effect on activities of daily living as measured with the Katz ADL index. Secondary outcomes are quality of life, mortality, nursing home admission, emergency department and out-of-hours General Practice (GP), surgery visits, and caregiver burden.

**Discussion:**

In a large, pragmatic trial conducted in daily clinical practice with frail older patients, several challenges and methodological issues will occur. Recruitment and retention of patients and feasibility of the interventions are important issues. To enable broad generalizability of results, careful choices of the design and outcome measures are required. Taking this into account, the U-PROFIT trial aims to provide robust evidence for a structured and integrated approach to provide care for frail older people in primary care.

**Trial registration:**

NTR2288

## Background

With an increasing number of older people in society, the number of frail older people with complex care needs will rise [1]. Frailty is a term often used among health care professionals to characterize older people who have a functional loss of resources in different domains. Frail older people have an increased risk for adverse health outcomes, such as mortality, morbidity and institutionalization [2-5]. The increasing number of frail older people will seriously challenge the health care system because primary care for these patients is currently fragmented, time consuming and reactive [6]. Because the care system does not address their needs, many older patients and their caregivers have a poor quality of life [7,8]. To preserve functional performance and maintain independent living in this vulnerable population, a transition is needed towards more proactive, integrated and structured health care for older people.

Until today, scientific evidence on how primary care providers can provide optimal care for frail older people with complex care needs is inconsistent. Previous intervention studies often used a selection of patients at risk combined with an additional geriatric assessment and follow-up visits [9,10]. However, evidence for these complex interventions is not clear. Moreover, it is unclear what the independent effectiveness of these interventions is.

One widely studied approach to select patients at risk is panel management. Panel management involves periodic reporting of clustered electronic medical record data from a certain 'patient panel' as an overview of the most important health parameters [11,12]. Missed patient encounters and care gaps can then easily be identified, which enables proactive, integrated and timesaving care. Panel management programs have been set up for various chronic diseases; however, integrated panel management approaches for frail older patients are lacking [13].

Other solutions to prevent functional decline are complex interventions, such as preventive home visiting programs with comprehensive geriatric assessments [9,14-16]. Little is known about the effectiveness of the different interacting components of these complex interventions. Elements that were demonstrated to be promising in different intervention studies are a multidisciplinary, multifactorial approach with tailor-made interventions and an individual assessment for frail older people provided by a (primary) care team with long-term follow-up [17-19].

To understand the effectiveness of these different approaches, we developed two interventions: a screening and monitoring intervention using routine healthcare data with the Utrecht Periodic Risk Identification and Monitoring system (U-PRIM) and a nurse-led multidisciplinary intervention program, U-CARE. In the **U**trecht **P**rimary care P**RO**active **F**railty **I**ntervention **T**rial (U-PROFIT), the effectiveness of the U-PRIM intervention, alone and in combination with U-CARE, will be assessed in comparison to usual care. The aim is to preserve physical functioning and improve quality of life for frail older people and their caregivers. The trial will be conducted from October 2010 to spring 2012. The aim of this paper is to describe the design of the U-PROFIT trial, the content of the two interventions and its methodological challenges.

## Methods

### Design and setting

**Figure 1:**
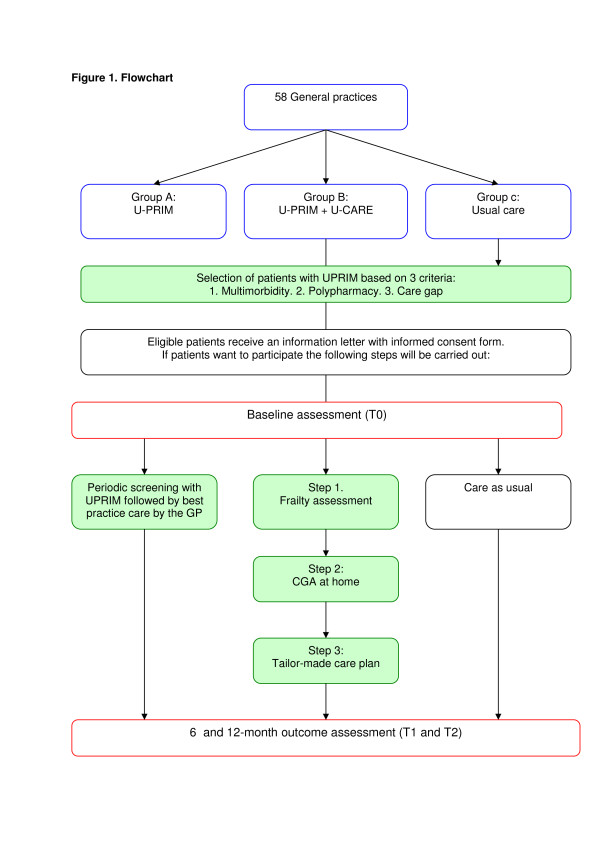
**Flowchart**.

A single-blind, three-armed, cluster-randomized controlled trial with a one-year follow-up is being conducted (see Figure 1). Recruitment was performed in three primary care networks with almost 70 practices in Utrecht, the Netherlands.

### Participants

#### Inclusion criteria

Selection of patients is performed by the U-PRIM system, a software application that is installed in all participating general practices. Exploring the electronic medical records (EMRs) in each general practice, U-PRIM will screen for three inclusion criteria in patients aged 60 years or older:

- Multimorbidity (defined as a frailty index score of ≥ 0.20; see the 'U-PRIM intervention' section)

- AND/OR

- Polypharmacy (defined as the chronic use of five or more different medications) [20]

- AND/OR

- Care gap in primary care of three or more years (defined as not having consulted the GP in the past three years, except for the yearly influenza vaccination).

#### Exclusion criteria

Terminally ill patients or patients living in an elderly home or nursing home are excluded. Reasons for exclusion are registered on the general practice level.

### Procedure

At the start of the inclusion period, U-PRIM automatically generates a list of frail patients of 60 years and older in every participating practice. Using the U-PRIM software, data extractions from the electronic medical records (EMRs) in the practices are uploaded to an external server area. Here, reports on frail patients are generated and delivered back to the general practice. To guarantee patient privacy, U-PRIM software encodes the personal data by means of a third trusted party procedure, so personal data are only disclosed to the general practice personnel.

Eligible patients are listed in the first U-PRIM report. These patients are approached by their GP with a patient information letter and informed consent form for participation in the U-PROFIT trial. In addition, patients are asked if they have an informal caregiver. If so, the caregiver is also invited to participate in the study to investigate caregiver burden.

In the practices in the control group, a similar U-PRIM report with potentially frail patients is generated, but this report is not visible to the GP.

### Ethical considerations

The U-PROFIT trial is approved by the Institutional Review Board of the University Medical Center Utrecht (UMCU) with protocol ID 10-149/O and registered in the Netherlands Trial Register: NTR2288.

### Randomization and blinding

The participating general practices are randomly allocated to one of the two intervention groups (A or B) or the control group (C) by cluster randomization on the general practice level (see flowchart). Practices in group A are allocated to the U-PRIM intervention, those in group B to the U-PRIM plus U-CARE intervention and the practices in group C formed the control group. Within the 58 participating general practices, clusters are created because some general practices are working closely together at the same location. Before randomization, clusters are stratified according to the expected number of frail older people in the general practice. The cluster size is estimated based on the number of invitations for the yearly influenza vaccination per practice.

### Blinding

#### Informed consent

A modified informed consent procedure is used to maintain a single-blind design; the so- called "consent to postponed information" [21,22]. With this procedure, a valid assessment of subjective outcomes can be obtained in a trial even if the patients cannot be blinded to the intervention. Additionally, selection bias and dropout in the control group can be reduced. In the U-PROFIT trial, patients were not informed as to which intervention group their general practice was allocated until the end of the follow-up period.

### Blinding of the GPs and practice nurses

Blinding the GPs and their practice nurses is not possible in this study because they are part of the intervention.

### Blinding the investigators

Because the investigators need to directly communicate with the general practices about the study, it is not possible to blind the investigators. However, during data analysis, investigators will be blinded to the data. When the data analysis is completed, this information will be disclosed to the investigators.

### The interventions

Two interventions are being tested in the U-PROFIT trial: 1. Screening and Monitoring of frailty (U-PRIM) and 2. Nurse-led multidisciplinary intervention program (U-CARE).

### Intervention 1: U-PRIM

The U-PRIM software application is an electronic monitoring system aiming at identification of older patients at increased risk of frailty in routine health care data. The software is based on periodic screening for relevant risk factors in the EMRs of the general practice.

U-PRIM screens for three core risk factors in patients aged 60 years or older. These are also the eligibility criteria of the U-PROFIT trial as described earlier (multimorbidity, polypharmacy and a care gap).

### Multimorbidity

The frailty index concept is used as an indicator of multimorbidity [23]. The frailty index uses 50 so-called 'health deficits': symptoms, signs, diseases, social problems and functional impairments, all routinely encoded in the EMR using International Classification of Primary Care (ICPC) codes (see Additional file 1). In the choice of the deficits, we followed previously published guidelines for the construction of a frailty index [24].

U-PRIM assesses the number of deficits in each individual. The frailty index score expresses the number of deficits present as a proportion of the total number of deficits [25]. Thus, a patent with 15 deficits has a frailty index score of 0.30 (15/50). For this study, multimorbidity based on the frailty index alone is defined as a frailty index score of ≥ 0.20 [26].

### Polypharmacy

The U-PRIM software screens the medication list for chronic drug use, using anatomical therapeutic chemical (ATC) codes. Chronic use is present when the medication was prescribed at least three times in the past year, with at least one prescription in the last six months. Polypharmacy is in this study is defined as 5 or more different drugs in chronic use in the past year [20].

### Care gap

The period that patients are out of sight of their GP is assessed to include possible care avoiders prone to self-neglect, for example patients with dementia, psychiatric conditions or alcohol abuse [27]. For this study, a "care gap" is defined as a period of at least 3 years without GP consultation, excluding the annual influenza vaccination.

### The U-PRIM procedure

In the U-PROFIT trial, the periodic U-PRIM frailty screening of the trial population takes place every three months in intervention groups A and B. This results in a U-PRIM report for each general practice with a selection of older patients at high risk of adverse health outcomes. Patients are prioritized by means of the frailty index score, with possibilities to prioritize according to polypharmacy or care gap. For an example of a U-PRIM report, see Additional file 2.

The report will be passed on to the GP in intervention groups A and B. In group A, GPs are asked to act upon the U-PRIM report in accordance with current available guidelines and best practices and to carry out interventions among the frail elderly patients if needed. In group B, all patients selected by U-PRIM will receive the additional steps of the U-CARE program (see intervention 2). In every participating practice in group A and B, a staff member is responsible for generating the reports with the U-PRIM computer program and for distributing the report among the care providers involved. These contact persons received protocolized, one-on-one guidance with the first U-PRIM report, with an explanation of the software application and suggestions on how to implement the report in daily clinical practice.

### Intervention 2: U-CARE program

U-CARE is a nurse-led, multidisciplinary intervention program to be used in frail patients selected by U-PRIM. Specially trained, registered practice nurses provide structured and integrated care based on a patients' needs approach.

U-CARE is developed by a multidisciplinary team consisting of researchers and practitioners in nursing and primary care medicine. Three experienced practice nurses, a panel of experts and a panel of older people are involved to validate the content.

The program consists of three steps. The first step is a frailty assessment for patients at risk. The second step is a comprehensive geriatric assessment (CGA) at home of frail patients. The third step is a tailor-made care plan with evidence-based interventions developed by the practice nurse. Details of the development and the content of the program are described elsewhere [Bleijenberg et al: Development of a nurse-led multidisciplinary intervention program (U-CARE) to prevent functional decline in frail older people in primary care, Unpublished].

### Step 1. Frailty assessment

The level of frailty in patients at risk selected by U-PRIM will be further explored with the Groningen Frailty Indicator questionnaire (GFI). The GFI is a 15-item validated questionnaire that assesses frailty from a functional ADL/IADL perspective on four domains: physical, cognitive, social and psychological [28]. Scores on each item are zero or one, and the total score ranges from 0 (not frail) to 15 (severely frail). We chose a score of 4 or higher as the relevant cut-off [29] for the selection of patients that should be visited for a comprehensive geriatric assessment. The GFI has shown high internal consistency and construct validity [30]. This questionnaire will be sent to all patients selected by U-PRIM.

The INTERMED for the Elderly (IM-E) [31] and the Groningen Wellbeing Indicator (GWI) are additional assessments included in U-CARE to enable a multidimensional approach and to measure patients' needs and complexity of care among frail patients on the GFI.

### Step 2. Comprehensive Geriatric Assessment at home (CGA)

For those patients identified as being frail, a CGA at home is conducted by a registered practice nurse. During this home visit, the practice nurse focuses on patients' health problems and needs in a structured manner based on the outcome of the frailty assessment. Based on the literature and their prevalence [32-34], ten health problems in older patients with additional assessments are included in the CGA (see Additional file 3).

### Step 3. Tailor-made care plan

In collaboration with the GP, the practice nurse will prepare a tailor-made care plan based on the outcome of step 2. This tailor-made care plan consists of interventions derived from evidence-based care plans developed by the research team, practice nurses and experts. For all ten health problems assessed in the CGA, separate evidence-based care plans are developed. The use of the care plan ensures uniformity among practice nurses in tailoring and delivering interventions per health problem. Flowcharts with suggested (nursing) interventions per health problem are developed as a practical tool and will help to guide the practice nurses through a structured process of decision making.

### Training program

All practice nurses will receive an extended U-CARE training program that consists of 5 weeks of 4 hours of lessons in class and 4 hours of self-study. During this training program, the included frailty assessments, the content of the CGA and the evidence-based care plans will be discussed. The U-CARE training program is set up in collaboration with the University of Applied Science Utrecht in the Netherlands.

One month prior to the start of the trial, all GPs and registered practice nurses from intervention group are participating in a training session of 4 hours in which the content of U-CARE program is explained and discussed. Additionally, a workshop about collaboration between GP's and practice nurses is set up.

### Outcomes and measurements

#### Primary outcome

The primary outcome of the U-PROFIT trial is the level of Activities of Daily Living (ADL) as measured with the Katz ADL index score [35]. The Katz index measures independence of ADL on six items (bathing, dressing, toileting, transferring, eating and the use of incontinence materials). The score ranges from 0 (total independence) to 6 (total dependence), and it is widely used to assess activities of daily living [36]. Baseline ADL functioning (T0) will be compared with ADL functioning after six months (T1) and one year of follow-up (T2). The questionnaire will be filled in by the patient or a proxy relative.

#### Secondary outcomes

Secondary outcome parameters will be measured at the same time as the primary outcome parameter (T0-T1-T2).

1. Quality of life will be measured with RAND-36 and EuroQol (EQ-5D) [37,38]

2. Mortality

3. Number of nursing home admissions

4. Number of emergency department and out-of-hours GP surgery visits

5. Caregiver burden measured with Self-Rated Burden (VAS) and Carer-Qol [39]

### Additional data collection

Routine health care data will be extracted from the EMRs of the participating practices. Socio-demographic data, such as age, gender, educational level, ethnicity, marital status and living situation, will be gathered at baseline. General practice characteristics, such as size, percentage of older people, working experiences and geographical location of the general practice, will also be gathered.

### Process evaluation

To understand the different components, their interaction and the applicability of the U-CARE program, a feasibility study will be conducted among doctors and practice nurses of intervention group B. Furthermore, interventions delivered by the practice nurse or other health care providers will be registered to gain insight into targeted interventions that are performed by the practice nurses.

The U-PRIM system will be evaluated on psychometric properties, prognostic value for adverse health outcomes and in concordance with the GFI, and the system will be refined following a user demands study.

In addition, qualitative data on patients' satisfaction with the U-CARE program will be qualitatively assessed. In the end, various data will be collected to perform a cost-effectiveness analysis, e.g., data on workload of the GP and practice nurses and time registration.

### Sample size calculation

At present, a valid estimation of the variance in the KATZ ADL results within and between general practices cannot be given because these data are not available for Dutch populations. For that reason, a formal power analysis for the cluster-randomized trial is not possible. Therefore, it is also not feasible in this study to take into account a potential cluster effect. In line with Faber et al., [40] we assume that any randomization effect per practice will be absent. Furthermore, we assume that with an expected number of at least 5000 frail older people included, relevant effects can be found on the outcome between the clusters because the power of a trial increases if the number of clusters, subjects, or repeated measures within a subject increases.

### Data analysis

The data will be analyzed using SPSS version 17.0. An 'intention to treat' analysis will be carried out to assess the differences between the intervention groups and the control group regarding ADL functional status. The variations in outcome between the groups will be calculated using mixed linear model analysis. Regression analyses and (co)variation analyses will be carried out when relevant to correct for baseline differences between older people in the three groups. Survival analysis using a Cox regression model with Kaplan-Meier survival curves will be used on mortality and admission into nursing homes. As social economic status (SES), gender, age and education are assumed to be potential effect modifiers, subgroup analysis will be applied where relevant. We will also take the working experience of the participating GPs and practice nurses into account in separate analyses.

## Discussion

In this paper, we present the research design and methodology of the U-PROFIT trial. This trial assesses the effectiveness of two interventions: a proactive screening and monitoring system and a nurse-led intervention program. U-PROFIT is unique because of the robust and pragmatic study design directly embedded in primary care practice, which maximizes the generalizability of the results. The integration of the U-PRIM proactive screening tool with the U-CARE nurse-led multidisciplinary intervention program, once proven effective, will provide an innovative, practical panel management approach for frail older people that can be broadly implemented in daily clinical practice. We met several challenges during the design and implementation of the U-PROFIT trial.

### Design

As mentioned, the two interventions are tested and embedded in routine clinical practice. Therefore, it s hard to create controlled experimental circumstances. We randomized on a practice level, and some practices may have already use screening lists or structured plans to provide care for older people, while others have not. In addition, in some practices, a practice nurse may have already been part of the practice team. Because all practices can be randomized in one of the intervention groups or in the control group, we consider these differences in elderly care at baseline as normal variations in clinical practice. In this way, both interventions are compared to the broad range of routine clinical care, enabling generalizability.

We chose a three-armed design for several reasons. First, our baseline assumption is that the U-PRIM screening followed by usual care and the combination of U-PRIM and U-CARE will both give better results than current usual care. Additionally, we hypothesize that both interventions are synergistic and that the effect of U-PRIM and U-CARE is more effective than the U-PRIM intervention alone [41].

### Outcome

The effectiveness of the interventions should be assessed on outcomes that are directly relevant for patients and their caregivers. We decided to take ADL functioning as measured with the Katz ADL index as the primary outcome. ADL functioning is generally reported as the most important parameter in the lives of older people [3]. The Katz ADL index is widely used in studies of prognosis and effects of treatments [36,42].

Additionally, a broad array of relevant secondary outcomes will be assessed to evaluate both interventions. These will be measured based on a combination of self-report, proxy report and data extraction out of routine healthcare data.

### Recruitment and compliance

Proper recruitment of older people for a clinical trial is often considered as complex [43,44]. To improve generalizability, it is important that not only healthy people are included but also less fit older people [3]. For logistical reasons, we opted for a postal approach of eligible patients by the participating GPs. In this approach, we tried to find the optimal balance between extensive information provision, which is strongly advised by the Institutional Medical Ethic Committee, and the need for short and simple information letters in this population. Although patients can contact their GP or the researchers for extra clarification, this postal approach might lead to some response bias with fewer cognitively impaired or frailer patients included than with a personal approach. To limit this problem, patients who do not give consent are approached by telephone two weeks after the information letter is sent, and home visits by a research assistant are offered.

Limiting informative censoring is a second challenge in elderly research. Informative censoring occurs when drop-outs happen for reasons directly related to the primary outcome [45]. In U-PROFIT, this can occur because frailer patients are more likely to die before we can evaluate functional status at the end of follow-up. To limit this problem and assess the extent of it, reasons for withdrawal will be collected, and an intention-to-treat analysis will be performed. Additionally, various retention strategies will be applied, e.g., home visits, interviews by phone when a postal questionnaire is difficult, small incentives, such as a U-PROFIT pen, and a newsletter to keep patients informed about the project.

### Development of the U-PRIM system

The U-PRIM system uses criteria that are known from literature to be linked to frailty, disability and morbidity and that have been selected by a local GP focus group as relevant in daily clinical practice [2,46,47]. Small pilot studies have shown that the current U-PRIM criteria identify a significant number of patients at high risk for frailty. However, the psychometric properties of U-PRIM and exact cut-off values for clinically relevant risk groups still have to be further assessed. The influence of EMR data quality on the U-PRIM output should also be examined [48].

While preparing for the U-PROFIT trial, major effort was put into building the software, implementing the U-PRIM system and testing it. However, during the trial, technical aspects of the U-PRIM system may need to be adjusted.

This might influence the current system of use and acceptance during the trial. We will assist participating centers by means of manuals, ICT assistance, and proactive contact after report generation to check for any content related questions or user feedback. With updates on the practical implications of ongoing U-PRIM research, we hope to keep all participating primary care providers on board. In this way, the U-PRIM system can be further developed into an easy-to-use frailty screening instrument that contributes to efficient and proactive panel management care. Requiring only sound EMR registration habits and periodic data upload, the U-PRIM system is an ideal candidate for efficient risk stratification of older people in primary care.

### Feasibility and adherence

The U-CARE program is a complex, multifactorial intervention with multiple components. In the trial, U-CARE will be provided by over 20 practice nurses and over 100 doctors, and optimal implementation is vital. By means of an extended training program and ongoing education during the trial, we aim for a uniform baseline level of knowledge and skills among the practice nurses. However, motivation for proactive care provision and professional experience with older patients can be different within the group of GPs and practice nurses. These differences reflect daily clinical practice, so general conclusions about the effectiveness can be drawn. However, the effectiveness may differ in relation to characteristics of health care professionals. For that reason, we will perform subgroup analyses.

Finally, this program is based on a proactive care approach. Some patients will appreciate the active interference of care providers, but other patients might not and consider it as patronizing. Possible benefits of a proactive outreach should therefore clearly outweigh the unwanted burden it may put on others.

### Strengths

Despite many challenges, we think that U-PROFIT offers many opportunities. First, the design of a three-armed, cluster randomized trial enables us to investigate the effectiveness of both interventions separately as well as in combination. Secondly, current literature recommends that trials on frailty should target persons aged 70 and older, because in younger age groups, frailty prevalence is thought to be too low [3]. However, during the development of U-PROFIT, general practitioners suggested to lower the age threshold for inclusion to 60. A substantial part of the ageing population in the practices consists of first generation immigrants of non-Dutch origin. In these elderly individuals, who often came to Holland for physical labor, frailty is reported to appear at a relatively young age [7]. With the inclusion of patients aged 60 years and older in our study, we include the group most relevant in current clinical practice.

The frailty index score is demonstrated to be a valuable indicator of the 'frailty state' of an individual. Frailty indices constructed differently, with different deficit content and considering different numbers of deficits, yield closely related results [25]. In this trial, we aim to demonstrate that the frailty index can be used for structured risk assessment in primary care practice, using routine care data.

For optimal implementation of the U-CARE intervention, we will maintain a training and supervision process of the practice nurses during the trial. In monthly meetings, special attention will be paid to collaboration between nurses and GPs to achieve optimal functioning of this important team. In addition, lectures and education about geriatric health problems will be performed. During regular project meetings, research updates will be provided to inform nurses and GPs.

While the intervention in non-pharmacological intervention studies is often poorly described, the interventions in the U-PROFIT trial consist of well-defined and thoroughly designed components. This will safeguard the reproducibility of the intervention program once the effectiveness is established.

Although various challenges have to be addressed, the U-PROFIT trial offers excellent opportunities for a valid scientific evaluation of a structured and integrated approach to improve physical functioning in frail older people in primary care. Once proven effective, it can be broadly implemented in daily clinical practice.

### Trial status

Data collection started in October 2010 and will end in March 2011. The results of this study will be expected in spring 2013.

## Competing interests

The authors declare that they have no competing interests.

## Authors' contributions

NB and ID drafted this manuscript. MN, MS and NW designed the study and wrote the grant application for the U-PROFIT trial. All authors are actively involved in the study and approved the final draft of this manuscript.

## Pre-publication history

The pre-publication history for this paper can be accessed here:

http://www.biomedcentral.com/1471-2318/12/16/prepub

## Supplementary Material

Additional file 1 **ICPC encoded frailty index deficits**. Click here for file

Additional file 2 **Lay-out of UPRIM report**. Click here for file

Additional file 3 **Overview of health problems, assessments and summary of interventions**. Click here for file
