# Supplementary material for: Proactive and integrated primary care for frail older people: design and methodological challenges of the Utrecht primary care PROactive frailty intervention trial (U-PROFIT)
Source: BMC Geriatr. 2012 Apr 25;12:16. doi: 10.1186/1471-2318-12-16 (PMC3373372; doi:10.1186/1471-2318-12-16)
Supplement: Additional file 1 — ICPC encoded frailty index deficits. [file 1471-2318-12-16-S1.PDF]

Additional file 1. ICPC encoded frailty index deficits

Deficit	ICPC*	ICPC-Label	Days**
1	K78	Atrial fibrillation/flutter	365
2	P74	Anxiety disorder/anxiety state	365
3	R96	Asthma	-
4	K77	Heart failure	-
5	T90	Diabetes mellitus	-
6	N88	Epilepsy	-
7	S70	Herpes zoster	365
8	S97	Chronic ulcer skin	365
9	D94	Chronic enteritis/ulcerative colitis	-
10	N89	Migraine	365
11	U99	Urinary disease, other	-
12	K88	Postural hypotension	365
13	L95	Osteoporosis	-
14	R81	Pneumonia	365
15	S91	Psoriasis	-
16	L88	Rheumatoid arthritis / related condition	-
17	P17	Tobacco abuse	-
18	P06	Sleep disturbance	365
19	N87	Parkinsonism, Parkinson’s disease	-
20	P15	Chronic alcohol abuse	-
20	P16	Acute alcohol abuse	365
21	A01	Pain general/multiple sites	365
21	A04	Weakness/tiredness general	365
21	A05	General deterioration	365
21	P78	Neuraesthenia/surmenage	365
22	B80	Iron deficiency anaemia	365
22	B81	Anaemia, Vitamin B12/folate def.	365
22	B82	Anaemia other/unspecified	365
23	L89	Osteoarthrosis of hip	-
23	L90	Osteoarthrosis of knee	-
23	L91	Osteoarthrosis other / related condition	-
24	P20	Memory / concentration / orientation disturbance	365
24	P70	Dementia / Alzheimer’s disease	-
24	P85	Mental retardation	-
25	R91	Chronic bronchitis / bronchiectasis	-
25	R95	Chronic obstructive pulmonary disease	-
26	K89	Transient cerebral ischaemia	365
26	K90	Stroke/cerebrovascular accident	-
27	P03	Feeling depressed	365
27	P76	Depressive disorder	365
28	K02	Pressure/tightness of heart	365
28	R02	Shortness of breath/dyspnoea w/o K02	365
29	N17	Vertigo/dizziness	365
29	H82	Vertiginous syndrome / labyrinthitis	365

30	L72	Fracture: radius/ulna	365
30	L73	Fracture: tibia/fibula	365
30	L74	Fracture: hand/foot bone	365
30	L75	Fracture: femur	365
30	L76	Fracture: other	365
31	H84	Presbyacusis	-
31	H85	Acoustic trauma	-
31	H86	Deafness	-
32	T05	Feeding problem of adult	365
32	T07	Weight gain	365
32	T08	Weight loss	365
32	T82	Obesity	-
32	T83	Overweight	-
33	K86	Hypertension uncomplicated	365
33	K87	Hypertension complicated	-
34	K74	Angina pectoris	365
34	K75	Acute myocardial infarction	365
34	K76	Other / chronic ischaemic heart disease	-
35	D17	Incontinence of bowel	-
35	U04	Incontinence urine	-
36	D72	Viral hepatitis	-
36	D97	Cirrhosis / liver disease NOS	-
37	A79	Malignancy NOS	
37	B72	Hodgkin's disease	-
37	B73	Leukaemia	-
37	B74	Malignant neoplasm blood other	-
37	D74	Malignant neoplasm stomach	-
37	D75	Malignant neoplasm colon/rectum	-
37	D76	Malignant neoplasm pancreas	-
37	D77	Malig. neoplasm digest other/NOS	-
37	F74	Neoplasm of eye/adnexa	-
37	H75	Neoplasm of ear	-
37	K72	Neoplasm cardiovascular	-
37	L71	Malignant neoplasm musculoskeletal	-
37	N74	Malignant neoplasm nervous system	-
37	R84	Malignant neoplasm bronchus/lung	-
37	S77	Malignant neoplasm of skin	-
37	T71	Malignant neoplasm thyroid	-
37	U75	Malignant neoplasm of kidney	-
37	U76	Malignant neoplasm of bladder	-
37	U77	Malignant neoplasm urinary other	-
37	X75	Malignant neoplasm cervix	-
37	X76	Malignant neoplasm breast female	-
37	X77	Malignant neoplasm genital other (f)	-
37	Y77	Malignant neoplasm prostate	-
37	Y78	Malignant neoplasm male genital / mammae	-
38	P18	Medication abuse	365
38	P19	Drug abuse	365

39	N86	Multiple sclerosis	-
39	N94	Peripheral neuritis/neuropathy	-
39	N99	Neurological disease, other	-
40	F83	Retinopathy	-
40	F84	Macular degeneration	-
40	F92	Cataract	-
40	F93	Glaucoma	-
40	F94	Blindness	-
41	P71	Organic psychosis other	365
41	P72	Schizophrenia	-
41	P73	Affective psychosis	365
42	K91	Atherosclerosis	-
42	K92	other PVD	-
42	K99	Cardiovascular disease other	-
43	T85	Hyperthyroidism/thyrotoxicosis	365
43	T86	Hypothyroidism/myxoedema	365
44	X87	Uterovaginal prolapse	-
44	Y85	Benign prostatic hypertrophy	-
45	K93	Pulmonary embolism	365
45	K94	Phlebitis/thrombophlebitis	365
46	D84	Oesophagus disease	365
46	D85	Duodenal ulcer	365
46	D86	Peptic ulcer other	365
47	A06	Fainting/syncope	365
47	A80	Trauma/injury NOS	365
48	A28	Limited function/disability NOS	-
48	B28	Limited function/disability	-
48	D28	Limited function/disability (d)	-
48	F28	Limited function/disability (f)	-
48	H28	Limited function/disability ear	-
48	K28	Limited function/disability (k)	-
48	L28	Limited function/disability (l)	-
48	N28	Limited function/disability (n)	-
48	P28	Limited function/disability (p)	-
48	R28	Limited function/disability (r)	-
48	S28	Limited function/disability (s)	-
48	T28	Limited function/disability (t)	-
48	U28	Limited function/disability urinary	-
48	X28	Limited function/disability (x)	-
48	Y28	Limited function/disability (y)	-
48	Z28	Limited function/disability (z)	-
49	Z12	Relationship problem with partner	365
49	Z14	Partner illness problem	365
49	Z15	Loss/death of partner problem	-
50	Z01	Poverty/financial problem	365
50	Z03	Housing/neighbourhood problem	365
50	Z04	Social cultural problem	365
50	Z29	Social problem NOS	365

* Dutch ICPC-1 version as currently in use in general practices

** ‘365 days’ indicates that the belonging ICPC code is only considered present when registered at least once in the past year. For ICPC codes without the ‘365 days’ indication, all time presence is considered.
