# Supplementary material for: Proactive and integrated primary care for frail older people: design and methodological challenges of the Utrecht primary care PROactive frailty intervention trial (U-PROFIT)
Source: BMC Geriatr. 2012 Apr 25;12:16. doi: 10.1186/1471-2318-12-16 (PMC3373372; doi:10.1186/1471-2318-12-16)
Supplement: Additional file 2 — Lay-out of UPRIM report. [file 1471-2318-12-16-S2.PDF]

Additional file 2. Lay-out of UPRIM report

Patient	Sex	Age	FI-score	Multimorbidity	Polypharmacy	Care Gap
Smith	F	87	0,26	13	12	5
Jones	M	63	0,22	11	16	18
Taylor	F	70	0,20	11	8	3
Brown	F	75	0,20	10	10	77
Smith	M	81	0,16	8	5	330
Johnson	F	72	0,14	7	6	32
White	F	94	0,08	5	4	1503
