# Supplementary material for: Proactive and integrated primary care for frail older people: design and methodological challenges of the Utrecht primary care PROactive frailty intervention trial (U-PROFIT)
Source: BMC Geriatr. 2012 Apr 25;12:16. doi: 10.1186/1471-2318-12-16 (PMC3373372; doi:10.1186/1471-2318-12-16)
Supplement: Additional file 3 — Overview of health problems, assessments and summary of interventions. [file 1471-2318-12-16-S3.PDF]

Additional file 1. Overview of health problems, assessments and summary of interventions

Health Problem	Assessment	Interventions and recommendations (summary)	Level of evidence*
1. Falls & Mobility	Get-up and Go-test Falls Efficacy Scale (FES-NL)	- Multidisciplinary, multifactorial, health/environmental risk factor; Screening/intervention programs in the community;<br>- A program of muscle strengthening and balance retraining, individually prescribed at home by a trained health professional;<br>- Medication control and, if possible, withdrawal of psychotropic medication.	- A1<br>- A1<br>- A1
2. Physical functioning	Instrumental Activities of Daily Living (IADL scale Lawton & Brody	- Exercise programs that consist of muscle strengthening, balance retraining, endurance and flexibility;<br>- Motivation, feedback, patient education;<br>- Practice should reflect the opportunities that are available in the community.	- A1<br>- A1-B
3. Nutrition & Malnutrition	Short Nutritional Assessment Questionnaire (SNAQ-65) Mini Nutritional Assessment (MNA)	- Screening the nutritional status<br>- Systematic identification of nutrition problem<br>- Educating health care workers on the consequences of malnutrition	- A1<br>- A1<br>- A1
4. Cognitive decline	Mini Mental State Examination (MMSE) Clock Drawing	- Support, motivating activities on social interaction, cognitive and physical activities<br>- Individual programs focus on IADL problems<br>- Cognitive stimulation and training	- B<br>- B<br>- A1
5. Polypharmacy	Medication review assessment	- Multifactorial interventions are more effective that mono-interventions<br>- Tailored patient education, instruction, support, feedback and follow-up<br>- Tools and reminders for adherence	- A1<br>- A1<br>- A1
6. Mood & depression	Mini Mental State Examination (MMSE) Geriatric Depression Scale (GDS) Observation List early symptoms Dementia (OLD) Clock Drawing test	- Screening instruments as part of the intervention strategy<br>- Exercise interventions<br>- Collaboration with other disciplines is essential	- A1<br>- C<br>- A1
7. Loneliness	De Jong-Gierveld loneliness scale	- Adapted interventions to target patients<br>- Patient education, instruction, referral<br>- Knowledge of health care workers about referral possibilities	- A1<br>- A1<br>- C

8. Vision problems & hearing loss	Hearing Handicap Inventory for the Elderly-Screening (HHIE-S)	- Determine the cause of reduced vision<br>- General practitioners have important role in screening (vision)<br>- Knowledge about referral possibilities and environmental adaptations	- A1<br>- A1<br>- D
9. Urinary incontinence	Protection Amount Frequency, Adjustment, Body image (PRAFAB)	- Bladder training<br>- Pelvic floor muscles training<br>- Planned bladder	- A1<br>- A1<br>- A1
10. Caregiver burden	Experienced burden informal care (EDIZ)<br>Caregiver Strain Index (CSI)	- Ask for use of support. If rejected, ask for underlying reason<br>- Nurses can play an important role in case finding<br>- Multidimensional programs on physical and mental support	- D<br>- C<br>- A2

Legend:
*Level of evidence:
A1: Systematic review of at least two independently conducted studies of A2 level
A2: Well-designed, double blind, randomized controlled trial
B: Comparative studies not randomized but well-designed cohort or case/control analytic studies (preferably from more than one center or research group)
C: Observational studies, case series studies
D: Expert opinion
